# An ancient family of SelB elongation factor-like proteins with a broad but disjunct distribution across archaea

**DOI:** 10.1186/1471-2148-11-22

**Published:** 2011-01-21

**Authors:** Gemma C Atkinson, Vasili Hauryliuk, Tanel Tenson

**Affiliations:** 1 University of Tartu, Institute of Technology, Tartu, Estonia

## Abstract

**Background:**

SelB is the dedicated elongation factor for delivery of selenocysteinyl-tRNA to the ribosome. In archaea, only a subset of methanogens utilizes selenocysteine and encodes archaeal SelB (aSelB). A SelB-like (aSelBL) homolog has previously been identified in an archaeon that does not encode selenosysteine, and has been proposed to be a pyrrolysyl-tRNA-specific elongation factor (EF-Pyl). However, elongation factor EF-Tu is capable of binding archaeal Pyl-tRNA in bacteria, suggesting the archaeal ortholog EF1A may also be capable of delivering Pyl-tRNA to the ribosome without the need of a specialized factor.

**Results:**

We have phylogenetically characterized the aSelB and aSelBL families in archaea. We find the distribution of aSelBL to be wider than both selenocysteine and pyrrolysine usage. The aSelBLs also lack the carboxy terminal domain usually involved in recognition of the selenocysteine insertion sequence in the target mRNA. While most aSelBL-encoding archaea are methanogenic Euryarchaea, we also find aSelBL representatives in Sulfolobales and Thermoproteales of Crenarchaea, and in the recently identified phylum Thaumarchaea, suggesting that aSelBL evolution has involved horizontal gene transfer and/or parallel loss. Severe disruption of the GTPase domain suggests that some family members may employ a hitherto unknown mechanism of nucleotide hydrolysis, or have lost their GTPase ability altogether. However, patterns of sequence conservation indicate that aSelBL is still capable of binding the ribosome and aminoacyl-tRNA.

**Conclusions:**

Although it is closely related to SelB, aSelBL appears unlikely to either bind selenocysteinyl-tRNA or function as a classical GTP hydrolyzing elongation factor. We propose that following duplication of aSelB, the resultant aSelBL was recruited for binding another aminoacyl-tRNA. In bacteria, aminoacylation with selenocysteine is essential for efficient thermodynamic coupling of SelB binding to tRNA and GTP. Therefore, change in tRNA specificity of aSelBL could have disrupted its GTPase cycle, leading to relaxation of selective pressure on the GTPase domain and explaining its apparent degradation. While the specific role of aSelBL is yet to be experimentally tested, its broad phylogenetic distribution, surpassing that of aSelB, indicates its importance.

## Background

Elongation factors EF-Tu in bacteria and EF1A in archaea and eukaryotes bind and deliver aminoacyl-tRNA (aa-tRNA) to the ribosome. These are the universal components of the EF1 family, which also contains a number of paralogous subfamilies with more restricted taxonomic distributions. EF1 family members all share a common domain structure, of a GTPase (G) domain and two beta barrel domains II and III, which interact with aa-tRNA. Some EF1 subfamilies also have additional N terminal or C terminal domains, responsible for lineage-specific intermolecular interactions. A number of specialized subfamilies recognize and deliver specific tRNAs to the ribosome. Initiator tRNA is specifically recognized by the EF1 family member e/aIF2γ in eukaryotes and archaea [1]. In nematodes, two mitochondrial EF-Tus are specialized for aa-tRNAs that differ from the canonical cloverleaf tRNA structure comprised of a D arm, T arm, anticodon arm and accepter loop. Nematode mtEFTu1 specifically binds T armless tRNAs that are commonly found in metazoan mitochondria [2], while mtEFTu2 is specialized for D armless Seryl-tRNA, specifically recognizing serine amino acid as well as the tRNA body [3]. Members of the SelB subfamily, close relatives of e/aIF2γ [4] are responsible for binding and delivering selenocysteinyl-tRNA to the ribosome in selenocysteine-utilizing bacteria, archaea and eukaryotes. At least in the case of bacterial SelB, interaction is specific both to the nature of the tRNA itself and the attached amino acid [5].

The amino acid selenocysteine is structurally similar to cysteine, but contains selenium instead of sulfur, which forms a selenol instead of thiol group. It is chemically more reactive than cysteine [6], and is found in several enzymes (selenoproteins). Selenocysteine has its own tRNA (tRNA^Sec^), but it lacks a dedicated codon in the standard genetic code. Instead, cells employ a specialized mechanism for its integration at a recoded UGA stop codon. Sec-tRNA^Sec ^forms a complex with the selenocysteine-specific elongation factor SelB and GTP, which in turn binds a structural element in the mRNA (the selenocysteine insertion element, or SECIS). This binding is direct in bacteria, via a C terminal extension in SelB [7]. In eukaryotic and archaeal SelB (eSelB or EF-Sec), the C terminal extension is shorter and in eukaryotes interacts with the SECIS element via a separate protein, the SECIS binding protein (SBP) [8]. An SBP ortholog is not present in archaea, and a functionally equivalent factor has not been identified, the closest homolog being ribosomal protein S30 [9]. Selenoproteins and the selenocysteine incorporation machinery have a patchy distribution across the tree of life [10]. However, as they are found in eukaryotes, archaea and bacteria, this suggests they were present in the last common ancestor of all life, and were subsequently lost in various lineages [11].

Pyrrolysine is another unusual naturally occurring amino acid [12]. It is a lysine derivative, with a pyrroline ring linked to the lysine side chain. Like selenocysteine, it has a specialized tRNA and is incorporated in response to a termination codon, in this case the 'amber' stop codon UAG [13]. Pyrrolysine has a narrower distribution than selenocysteine, being utilized only in Methanosarcineae archaea and one bacterium, *Desulfitobacterium hafniense *[13].

In archaea, only some methanogenic archaea are known to utilize selenocysteine, and as such, the synthesis and incorporation machinery (selenophosphate synthetase (SelD), tRNA^Sec ^(SelC) and SelB) are only found in these organisms [10]. A paralog of archaeal SelB (aSelB) has been identified in Methanosarcinales, and proposed to be a hitherto unidentified pyrrolysine-specific elongation factor (EF-Pyl) [14]. A structure of the putative EF-Pyl was determined and deposited in the protein data bank in 2007 (PDB ID 2ELF[15]). However, bacterial EF-Tu has been found to be capable of binding archaeal Pyl-tRNA, suggesting that the archaeal ortholog of EF-Tu, aEF1A, may also be capable of delivering Pyl-tRNA to the ribosome without the need of a specialized factor [16].

We have identified EF-Pyl/aSelB-like homologs (herein referred to as aSelBL) in a range of archaea that is broader than the distribution of selenocysteine and pyrrolysine. We have carried out comparative phylogenetic and sequence analysis of the aSelBL subfamily. Strong structural similarity with aSelB and conservation of tRNA and ribosomal RNA interaction sites suggests that in some archaea, aSelBL may be a functional translation elongation factor. However, disrupted G domains in subgroups of aSelBL raise the possibility that the aSelB proteins are not typical molecular switches like classical translational GTPase elongation factors. Their taxonomic distribution, while non-continuous, is broad, and generally (although not exclusively) associated with methanogenic high-cysteine archaea. We discuss possible roles for these unusual proteins.

## Results

### Phylogenetic analysis

**Figure 1:**
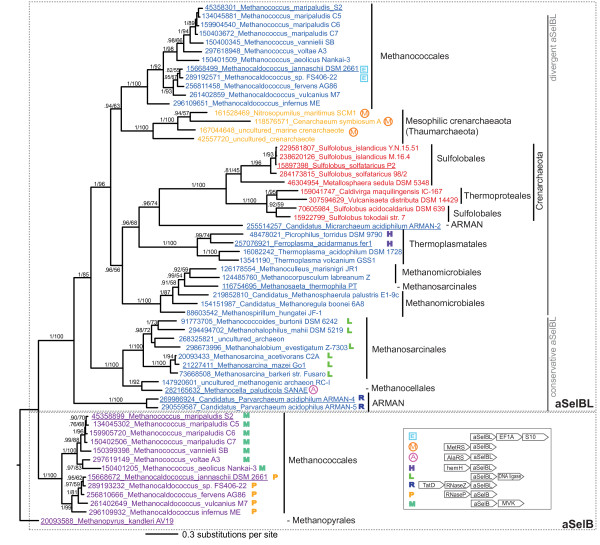
**Bayesian inference tree of aSelB and aSelBL from 311 aligned amino acid positions**. Bayesian inference posterior probability (BIPP) and maximum likelihood bootstrap percentage (MLBP) support are indicated on branches. Only branches with >.70 BIPP support are labelled with BIPP and MLPP values. The scale bar below the tree shows the evolutionary distance expressed as substitutions per site. The SDSF at the end of the MrBayes run was 0.005. Numbers in taxon names are NCBI GI numbers, and underlined names indicate those taxa included in the illustrative alignment (Figure 2). Archaea labelled ARMAN are those from the clade of "Archaeal Richmond Mine Acidophilic Nanoorganisms" [18]. Symbols following taxa names indicate aSelB/aSelBLs with conserved genomic context (Additional file 3). The key for genomic context symbols is in the inset box, showing the location of genes in relation to aSelBL or SelB. Gene names are abbreviated as follows: MVK, mevalonate kinase family protein; RNase P, Ribonuclease P; TatD, TatD-related deoxyribonuclease; RNase Z, Ribonuclease Z/Lactamase B family protein; hemH, Protoheme ferrolyase (ferrochelatase); alaRS, alanyl-tRNA synthetase; metRS, methionyl-tRNA synthetase.

**Figure 2:**
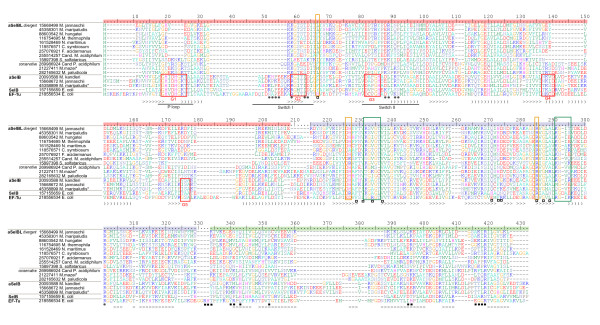
**Multiple sequence alignment of representative taxa across the aSelB/aSelBL tree**. Red boxes show the location of the GTP binding loops (G1-5). Green boxes show the location of 16S rRNA interaction sites in EF-Tu [28]. Residues in the core of the aminoacyl-binding pocket of aSelB are boxed in orange [27]. Symbols immediately beneath EF-Tu show sites that make contact with aminoacyl group (open squares), the acceptor stem (grey circles) and the T stem (filled squares) in the structure of tRNACys-EF-Tu [26]. The colored ruler shows the domains (red: GTPase, blue: domain II, green: domain III). The C terminal extension of aSelB is not shown. Symbols beneath the alignment show secondary structure based on aSelBL (PDB ID 2ELF). '>' is sheet and ')' is helix. Asterisked taxa names are those with a crystal structure (Figure 3).

Sequence searching revealed the presence of homologs of archaeal SelB in the genomes of many Euryarchaea, the recently designated phylum Thaumarchaeota [17], and Sulfolobales and Thermoproteales within Crenarcheatoa (Figure 1, Table 1, Additional files 1 and 2). Thus, aSelB homologs are present, although with a disjunct distribution, in all three major lineages of archaea [17]. ML and BI phylogenetic analysis of bacterial SelB, aSelB, EF-Tu and aIF2γ shows that the aSelB homologs form a monophyletic group with full support (BIPP 1.0, MLBP 100%, Additional file 1). 'True' aSelB (i.e. the previously identified and characterized aSelB selenocysteine elongation factor) from *Methanopyrus kandleri *represents a sister group to a clade that comprises 'true' aSelB from Methanococcales, plus a divergent clade henceforth referred to as aSelB-like (aSelBL). The monophyly of Methanococcale aSelB+aSelBL to the exclusion of *M. kandleri *aSelB is not strongly supported (0.88 BIPP, 68 MLBP). However, aSelBL is fully supported as monophyletic and is also supported by a 15 amino acid deletion in the GTPase domain, relative to "true" aSelB (alignment positions 34-57, Figure 2). Species of *Methanococcus *and *Methanocaldococcus *have both aSelB and aSelBL. Thus it appears that aSelBLs are not orthologs of aSelB, but are paralogs or xenologs, originating via gene duplication or lateral transfer, rather than speciation. Only *M. kandleri *has aSelB without a copy of aSelBL.

**Table 1:** Distribution of aSelBL, aSelB, SelD and pylS across archaeal full genomes

Taxonomic groupings*		Proteins identified*			
*Thaumarchaeota*	*Nitrosopumilus_maritimus SCM1*	**aSelBL**			

*Crenarchaeota*	*Desulfurococcales*	-			
	*Sulfolobales*				
	***Metallosphaera sedula DSM 5348***	**aSelBL**			
	***Sulfolobus acidocaldarius DSM 639***	**aSelBL**			
	***Sulfolobus islandicus***	**aSelBL**			
	***Sulfolobus solfataricus***	**aSelBL**			
	***Sulfolobus tokodaii str. 7***	**aSelBL**			
	*Thermoproteales*				
	***Caldivirga maquilingensis IC-167***	**aSelBL**			
	*Pyrobaculum (multiple species)*				
	*Thermofilum pendens Hrk 5*				
	*Thermoproteus neutrophilus V24Sta*				
	***Vulcanisaeta distributa DSM 14429***	**aSelBL**			

*Euryarchaeota*	*Archaeoglobales*	-			
	*Halobacteriales*	-		[SelD]**	
	*Methanobacteriales*	-			
	*Methanococcales*				
	***Methanocaldococcus fervens AG86***	**aSelBL**	aSelB	SelD	
	***Methanocaldococcus infernus ME***	**aSelBL**	aSelB	SelD	
	***Methanocaldococcus jannaschii DSM 2661***	**aSelBL**	aSelB	SelD	
	***Methanocaldococcus sp. FS406-22***	**aSelBL**	aSelB	SelD	
	***Methanocaldococcus vulcanius M7***	**aSelBL**	aSelB	SelD	
	***Methanococcus aeolicus Nankai-3***	**aSelBL**	aSelB	SelD	
	***Methanococcus maripaludis***	**aSelBL**	aSelB	SelD	
	***Methanococcus vannielii SB***	**aSelBL**	aSelB	SelD	
	***Methanococcus voltae A3***	**aSelBL**	aSelB	SelD	
	*Methanomicrobiales*				
	***Candidatus Methanoregula boonei 6A8***	**aSelBL**			
	***Methanocorpusculum labreanum Z***	**aSelBL**			
	***Methanoculleus marisnigri JR1***	**aSelBL**			
	***Methanosphaerula palustris E1-9c***	**aSelBL**			
	***Methanospirillum hungatei JF-1***	**aSelBL**			
	*Methanopyrales*				
	*Methanopyrus kandleri AV19*		aSelB	SelD	
	*Methanosarcinales*				
	***Methanococcoides burtonii DSM 6242***	**aSelBL**			pylS
	***Methanohalobium evestigatum Z-7303***	**aSelBL**			pylS
	***Methanohalophilus mahii DSM 5219***	**aSelBL**			pylS
	***Methanosaeta thermophila PT***	**aSelBL**			[pylS]**
	***Methanosarcina acetivorans C2A***	**aSelBL**			pylS
	***Methanosarcina barkeri str. Fusaro***	**aSelBL**			pylS
	***Methanosarcina mazei Go1***	**aSelBL**			pylS
	*Thermococcales*	-			
	*Thermoplasmales*				
	***Ferroplasma acidarmanus fer1***	**aSelBL**			
	***Picrophilus torridus DSM 9790***	**aSelBL**			
	***Thermoplasma acidophilum DSM 1728***	**aSelBL**			
	***Thermoplasma volcanium GSS1***	**aSelBL**			

*Nanoarchaeota*	*Nanoarchaeum equitans Kin4-M*	-			

*Orders that carry either aSelB or aSelBL are expanded to show species and strain (full data in Additional file 2).

**Square brackets indicate divergent or partial sequences (details in text).

Phylogenetic analysis of aSelB/aSelBL alone was carried out to reveal the internal branching structure of the subfamily, using more universally alignable homologous amino acid positions (Figure 1). *Candidatus Parvarchaeum acidiphilum ARMAN-4 *and *-5 *form a sister group to the remaining aSelBLs, which have strong BI and moderate ML support for monophyly (1.0 BIPP, 85% MLBP, Figure 1). The ARMAN (Archaeal Richmond Mine Acidophilic Nanoorganisms) bacteria are proposed to branch near the root of the Euryarchaeota [18]. This suggests that aSelBL may have originated within or before the lineage leading to Euryarchaeaota. However, one of the ARMAN bacteria, *Candidatus Microarchaeum acidiphilum ARMAN-2 *is nested deeply within aSelBL, grouping with the Crenarcheaota clade with moderate support (0.96 BIPP, 74% MLBP, Figure 1). Both the Thaumarchaeota and Crenarchaeota + *M. acidiphilum *clades are nested strongly within Euryarchaeota (1.0 BIPP, 96% MLBP, Figure 1). This does not agree with currently accepted deep archaeal phylogeny [17,19]. Thus, the aSelBLs of Thaumarchaeota, Crenarchaeota and *M. acidiphilum *appear to have arisen as a result of duplication and loss, or horizontal gene transfer (HGT) from Euryarchaea. Another possible case of HGT may have occurred in the lineage to *Methanosaeta thermophila*, which branches within the Methanomicrobiales with full BIPP and MLBP support, instead of its home clade of Methanosarcinales [17,19], which is otherwise fully supported as a monophyletic group (Figure 1).

BLASTP searches with pyrrolysine synthetase (PylS) confirmed that pyrrolysine synthesis is limited to the Methanosarcinales (Table 1). In one Methanosarcinale, *Mathenosaeta thermophila*, only a partial PylS sequence was identified from a short sequence read, as previously noted [20]. The presence of selenophosphate synthetase (SelD) in a genome is a signature for selenium utilization [10]. BLASTP searches with SelD confirmed that selenium utilization is limited to Methanococcales and Methanopyrus (Table 1). However one divergent SelD-like homolog was also found in *Haloarcula marismortui*, as previously identified [21].

### Sequence and structure of aSelBL

The most striking feature of the aSelBL subfamily alignment is a GTPase domain that deviates from the classical architecture, characterized by the presence of GTP-binding motifs (G1-G5) [22]. In particular, G1 (within the phosphate binding (P) loop), which binds the α- and β-phosphates of both GDP and GTP has experienced a 4-5 amino acid deletion in some aSelBLs relative to other EF1 family members (alignment positions 21-25, Figure 2).

The extent of the G domain disruption follows the phylogeny, with distinct clades in the tree having specific sequence patterns. The most conservative aSelBLs are the clades of *Candidatus Parvarchaeum acidiphilum ARMAN-4 *and *-5*, and Methanocellales + Methanosarcinales ("conservative aSelBL", Figure 1). These taxa retain some conserved residues of the G1 and G4 motifs, although with less overall sequence conservation in the motifs than SelB and aSelB (Figure 2). The monophyly of the more divergent aSelBLs ("divergent aSelBL", Figure 1) is well supported in the tree (1.0 BIPP, 96% MLBP, Figure 1), and independently supported by a 10-14 amino acid indel (alignment positions 140-149, Figure 2). This region is conserved in conservative aSelBL, aSelB and other EF1 family members, but deleted in divergent aSelBLs (Figures 1, 2). Similarly, the G1 (P loop) region contains a large deletion in the divergent aSelBLs (alignment positions 20-25, Figure 2), with the exception of the Crenarchaeota. The latter do not have the deletion, however sequence homology between Crenarchaeota and the other aSelBLs is ambiguous in this region, and it is possible that the lack of the deletion may actually correspond to a secondary insertion.

Despite the large deletions, regions within the G domain, specifically sequence motifs G2 and G3, are conserved in aSelBL, however often with different residues relative to aSelB (Figure 2). In P-loop GTPases, the threonine of the G2 "RGI**T**I" motif (within the switch I or the effector loop) and the glycine of G3 "DXP**G**H" motif (within switch II) bind the γ-phosphate of GTP, thus discriminating the nature of the bound nucleotide. Switches I and II are at the core of GTPase function, undergoing conformational changes between GTP and GDP binding states [23]. The "RGITI" G2 motif common to trGTPases is conserved at the 60% level in aSelBL as "KGTXX" (where X is any unconserved amino acid). Similarly, "DXPGH" of G3 has a 60% consensus of "E/DPXXX" in aSelBL. The histidine of G3 (position 86, Figure 2) is unconserved in aSelBL, although it is critical for GTP hydrolysis, and therefore universally conserved in other translational GTPases [24]. In EF-Tu, it is the positioning of His84 between Val20 of the P loop and Ile60 of Switch I by the sarcin/ricin loop (SRL) of the ribosome that activates the GTPase [24]. The differential conservation between aSelBL and other translational GTPases suggests that aSelBL has diverged in GTPase function, both in terms of GTP/GDP discrimination and GTP hydrolysis, but that the divergence is not due to a general degradation of the GTPase domain. The switch I and II regions are also involved in binding the tRNA acceptor stem, providing a physical link between tRNA and GTP binding to EF-Tu (Figure 2, [25,26]). Thus the functional divergence in aSelBL in switches I and II may have involved modification or refinement of tRNA-binding capabilities.

**Figure 3:**
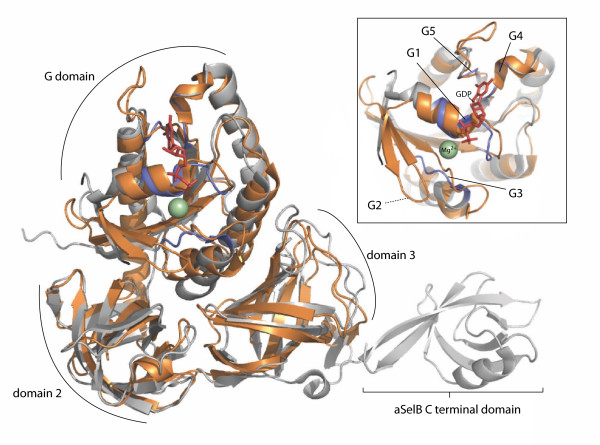
**Structural alignment of aSelBL and aSelB**. aSelBL ("EF-Pyl") from *Methanosarcina mazei *(PDB ID 2ELF) is shown in bronze, and aSelB from *Methanococcus maripaludis *in complex with GDP (PDB 1WB1) is shown in silver. Blue colouring indicates the location of the nucleotide binding motifs in aSelB. GDP and the magnesium ion bound to aSelB are shown as red sticks and a green sphere respectively. The inset box shows a more detailed view of the G domain, with the G1 and G3-5 motifs from aSelB labelled. As G2 is disordered in aSelB, the equivalent region in aSelBL is indicated.

One of the most conservative aSelBLs, *M. mazei *aSelBL has been subjected to X-ray analysis. Diffraction data were collected in 2004, and the aSelBL structure was determined and deposited in the protein databank in 2007 (PDB ID 2ELF) [15]. Structural alignment with aSelB [27] shows that the overall structures are remarkably similar (Figure 3). Indeed, even in the GTPase domain, the structures are completely superimposable, with the exception of the switch I region, which is disordered in aSelB [27]. The aSelBLs do not, however, carry the C terminal extension that is present in all other SelBs and is required for SECIS or SECIS-binding protein recognition (Figure 3). Thus, aSelBLs are unlikely to be able to respond to SECIS, or similar elements in the mRNA.

**Figure 4:**
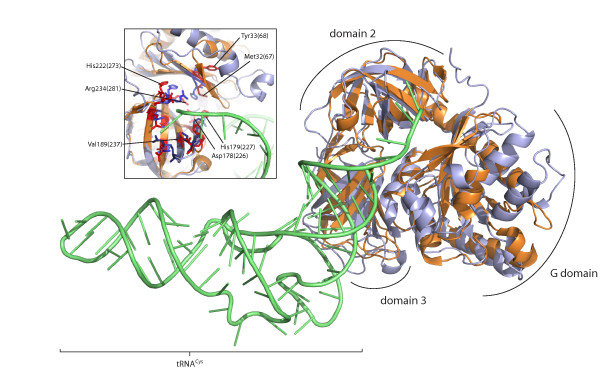
**Structural alignment of aSelBL and EF-Tu-Cys-tRNACys**. aSelBL ("EF-Pyl", PDB ID 2ELF) is shown in bronze and EF-Tu-Cys-tRNACys (PDB 1B23) is shown in pale blue and green, for EF-Tu and tRNA respectively. The inset box shows a more detailed view of the aminoacyl-binding pocket. Residues lining the pocket are shown in red for aSelBL, with equivalent EF-Tu sites in blue. Residue labels use *M. mazei *numbering, with Figure 2 alignment numbers in parentheses.

Domains II and III are much more conserved than the G domain across aSelB/aSelBL (Figure 2). Structural and mutational anlaysis shows that in *M. maripaludis *aSelB, the core of the aminoacyl-binding pocket is formed by residues Asp191, His192 and Arg247 [27] (orange boxes, Figure 2). In aSelBL, Asp191 and His192 (positions 226-227, Figure 2) are universally conserved, with the exception of the longest branched aSelBLs in the tree (Sulfolobales + *M. acidiphilum*), which lack His192. Arg247 (position 285, Figure 2) is universally conserved. Similarly, sites that make contact with aminoacyl group, the acceptor stem and (to a lesser extent) the T stem in the structure of tRNACys-EF-Tu [26] are often well conserved between aSelB and aSelBL (Figure 2). The structure of aSelBL can be superposed on that of the EF-Tu-Cys-tRNACys ternary complex [26], showing that the aminoacyl binding pocket is also structurally well conserved (Figure 4). Thus, aminoacyl-tRNA binding capabilities appear to be retained in aSelBL.

A particularly interesting feature of the aSelB structure is Phe51 (position 67, Figure 2), which protrudes into the aminoacyl binding pocket [27]. This phenylalanine, which can be replaced by tyrosine in some SelBs, is proposed to form a hydrophobic "lid" that protects selenocysteine from oxidation [27]. Such a lid may also be a feature of aSelBLs, which all carry a Phe or Tyr at position 67 and/or position 68 (Figure 2). Although Tyr33 (at position 68 in Figure 2) is facing away from the aminoacyl-binding pocket in the aSelBL structure (Figure 4), this residue is proposed to be flexible in aSelB [27] and its orientation could change upon aa-tRNA-binding.

The structure of aa-tRNA-EF-Tu on the ribosome reveals specific interactions between the elongation factor and ribosomal RNA [28]. Two patches in particular in domain II of EF-Tu associate with the shoulder domain of 16S rRNA [28]. Both of these patches are well conserved in aSelBL (green boxes, Figure 2), suggesting ribosome binding may also have been retained in aSelBL evolution.

### Genomic context

The genomic context of a gene of interest can provide clues about function, as functionally related genes are often co-located and co-transcribed in archaea as well as bacteria [29]. Pyrrolysine biosynthesis and utilisation genes cluster together [30], as do ribosomal RNA genes [31] and elongation factors in some archaea [32]. Genes flanking the *aSelBL *gene are mostly unconserved in context (Additional file 3). However there are some co-localizing genes that are transcribed in the same direction as *aSelBL *with no intervening gene on the complementary strand and are conserved in multiple species (Figure 1 Additional file 3). These encode genes with various functions, and in general, the significance (if any) of their colocalization with *aSelBL *is uncertain. Methanosarcinales carry an ATP-dependent DNA ligase upstream of *aSelB*, often following a CBS (Cystathionine Beta Synthase) domain-containing protein. The aSelBs of *Picrophilus torridus *and *Ferroplasma acidarmanus *are immediately downstream of ferrochelatase hemH, and downstream of two ribonucleases in *Candidatus Parvarchaeum acidiphilum ARMAN-4 *and *-5 *(Figure 1, Additional file 3). Other conserved flanking genes are translation-associated. Three of the four Thaumarchaea (*Nitrosopumilus maritimus*, *Cenarchaeum symbiosum *and uncultured marine crenarchaeote HF4000 APKG7F19) encode a methionyl-tRNA synthetase upstream of aSelB, and *Methanocella paludicola *carries an upstream alanyl-tRNA synthetase. Additionally, *Methanocaldococus Jannaschii *and *Methanocaldococcus sp. FS406-22 *aSelBs are upstream of the ribosomal protein S10 and elongation factor 1A.

## Discussion

### Origin of aSelBL

Given the wide distribution of aSelBL in methanogens, and its presence in all currently known species of Thaumarchaeaota, the original duplication of the ancestral aSelB appears to be very ancient, occurring very early in archaeal evolution, possibly before the archaeal last common ancestor (aLCA). However, the well supported nesting of Thaumarchaeote aSelBL within methanogens appears to suggest HGT. This is a surprising association, as it would require that the transfer occurred before the divergence of all currently sampled genera within the Thaumarchaeaota phylum. The presence of aSelBL in only Sulfolobales and two Thermoproteales of Crenarchaeota may be due to one or more horizontal transfers into Crenarchaeota. Alternatively, as there are only branches with weak to moderate support (0.94-0.96 BIPP, 56-74% MLBP) separating Crenarchaeota and Thaumarcaheota, aSelBL may have been inherited vertically from the ancestor of Crenarchaeota and Thaumarcaheota. In this case, loss of aSelBL must have occurred in the Desulforococcale lineage, and at least once more to account for its absense in *Pyrobaculum*, *Thermofilum *and *Thermoproteus *genera. Within Euryarchaeota, aSelBL was also lost or was never present in Halobacteriales, *Archaeoglobus*, Methanobacteriales, Methanopyrus (Table 1, Additional file 2). At present, it is impossible to retrace the origin of aSelBL in all archaea, but it is likely that both differential loss and HGT have been involved.

Assuming SelB was present in the LCA of archaea as previously suggested [11], classical aSelB must also have been lost in multiple lineages, as it is only found in *Methanococcales *and *Methanopyrus*. As aSelB has experienced more loss than aSelBL, the function of the latter seems more critical than the former in a number of archaea. Indeed, aSelBL is found in some of the smallest genomes of archaea, for example *Picrophilus torridus *(1.5 megabases) and *Methanocaldococcus infernus *(1.3 megabases), and also the largest (*M. acetovorans*, 5.8 megabases). This suggests that loss of aSelBL has not been due to general genome size reduction.

### Possible functions of aSelBL

Genomic context that includes genes for aminoacyl-tRNA synthetases, ribosomal protein S10 and EF1A suggests that at least in some cases, aSelBL may still be involved in some aspect of translation. Indeed the structure of conservative aSelBL is strikingly similar to aSelB and patterns of sequence conservation suggest that all aSelBLs are capable of binding the ribosome and aminoacyl-tRNAs. However, the major disruptions in the GTPase domain raise the question of whether aSelBL can function as a classical GTPase. Additional differences in the G domains *within *the aSelBL group also suggest that its function has diverged to some extent between conservative and divergent aSelBLs.

Although the aSelBLs arose from a selenocysteine-specific elongation factor, they are unlikely to have retained this role. Firstly, there is no C terminal extension with which to recognize the SECIS or SECIS-binding proteins. Secondly and more importantly, other components of the incorporation machinery are not present in most of the aSelBL-containing taxa (Table 1, [10]), and neither are selenoproteins [33]. aSelBL has previously been proposed to be a pyrrolysine-specific elongation factor in Methanosarcinales [14]. However, in archaea, only Methanosarcinales utilise pyrrolysine and encode the pyrrolysyl-tRNA synthetase gene (*pylS*, Table 1). Thus, as with the selenocysteine usage distribution, the pyrrolysine distribution is only a subset of the full aSelBL distribution.

Despite the weight of evidence suggesting that aSelBL is not a selenocysteine-tRNA elongation factor, the amino acids responsible for selenocystine specificity are well conserved in aSelBL. This suggests that aSelBL is still specific for a single amino acid, with the most likely candidate being cysteine, given its structural similarity to selenocysteine. The conserved, positively charged residues that line the aminoacyl binding pocket of aSelBL could potentially accommodate the negative thiol group of cysteine, just as they accommodate the negative selenol group in classical SelBs. Indeed, bacterial SelB cannot entirely distinguish sulfur from selenium, and is able to bind Cys-tRNASec tightly enough to allow for Cys-tRNASec delivery to the ribosome [34]. This raises the possibility that after duplication of the ancestral aSelB/aSelBL protein, aSelBL was recruited for a process requiring Cys-tRNA recognition, losing its SECIS or SBP-binding domain in the process. Following loss of selenium utilization, aSelB was lost from many archaea, but aSelBL was maintained in many lineages, and horizontally transferred to others.

This raises the question of what cysteine-associated mechanisms(s) aSelBL may be involved with. Cysteine is overrepresented in methanogenic archaea, which contain many iron sulfur cluster proteins, coordinating iron via cysteine-rich motifs [35]. Some methanogenic archaea lack the canonical class I cysteine-tRNA synthetase (CysRS). In these archaea, Cysteine is synthesized via a recently discovered tRNA-dependent pathway. O-phosphoseryl-tRNA synthetase (SepRS) ligates O-phosphoserine to tRNACys, which is converted to Cys-tRNACys by Sep-tRNA:Cys-tRNA synthase (SepCysC) [36,37]. It has been suggested that the tRNA-dependent cysteine biosynthesis pathway is a strategy to protect cysteine from thermal degradation, by limiting the amount of free cysteine [38]. Indeed, there is a correlation between the presence of SepRS and high cysteine content proteomes [39]. Potentially any cysteinyl-tRNA binding protein may aid in this protective function. Therefore the role of aSelBL may be primarily to bind and protect the highly labile cysteine from degredation. Consistent with this, the protective hydrophobic "lid" of the aminoacyl-binding pocket of aSelB that could protect selenocysteine/cysteine from oxidation also appears to be present in aSelBL. There is an association of aSelBL with high cysteine methanogens (Additional files 2 and 4). However, this clustering may simply be due to the common ancestry of methanogens, as the non-methanogens (Thermoplasmatales, Sulfolobales and Thaumarchaeota) that also encode aSelBL do not show a similar enrichment in cysteine (Additional file 4).

With its conservation of ribosome, tRNA and aminoacyl-binding sites, aSelBL may be a specialized elongation factor for incorporation of cysteine or another amino acid. Classical EF1 family elongation and initiation factors bind to the ribosome in complex with aa-tRNA and GTP, with complex formation between the GTPase and aa-tRNA strongly promoted in the presence of GTP [40-42]. In accordance with this paradigm, formation of the bacterial SelB*Sec-tRNA^Sec^*GTP ternary complex is strongly promoted by GTP [5]. However, when tRNA^Sec ^is charged with an amino acid other than selenocysteine, i.e. serine, which acts as a precursor in selenocysteine biosynthesis [43], the interaction becomes insensitive to G nucleotides, effectively decoupling the GTPase function from the tRNA binding function [5].

This behaviour of bacterial SelB invites us to speculate on possible scenarios for the functional evolution of aSelBL. Following duplication of aSelB, the redundant aSelBL may have acquired a new aa-tRNA partner. This would have led to loss of the GTP-dependence of ternary complex formation, due to the absolute selenocysteine specificity of the parental SelB [5]. Loss of the functionality of the GTPase cycle would have led to a relaxation of selective pressure on the G domain in terms of GTP/GDP discrimination, but continued selection for aa-tRNA-binding capabilities, resulting in an aSelBL that retains the classical GTPase fold (Figure 3). The conserved motifs G1-5 of the G domain are poorly conserved in aSelBL, including the histidine of switch II that is critical for GTP hydrolysis [24]. Thus aSelBL appears unlikely to hydrolyse GTP on the ribosome via the same mechanism as other proteins of the translational GTPase superfamily. However, the functionality of the aSelBL G domain needs to be addressed experimentally, as these proteins may still hydrolyse GTP or another nucleotide via a hitherto unknown mechanism. Alternatively, they may be non-enzymatically delivering aa-tRNA to the ribosome. This would not be without precedent, as a eukaryotic factor has recently been discovered that delivers aa-tRNA to the ribosome independently of GTP [44]. We also can not exclude the possibility that aSelBL may be involved in an aspect of tRNA biochemistry not directly related to protein synthesis. There is a wide variety of tRNA processing mechanisms in archaea [45]. For example, tRNA splicing, reassembly of split tRNA genes, the addition of a G-1 residue in tRNA^His ^and adding the 3' CCA end, the three bases required for aminoacylation.

## Conclusions

Although the function of aSelBL remains obscure, the wide distribution of these proteins among diverse archaea suggests that they have functions of some significance. This important enzyme has so far been overlooked experimentally, but future characterization may reveal important mechanisms of translational control in archaea.

## Methods

Sequences were retrieved by BLASTP searches against archaeal genomes in NCBI genomes (Additional file 2). *Methanosarcina Mazei *("EF-Pyl"), *Methanospirillum hungatei *and *Vulcanisaeta distributa *aSelBLs were used as queries, with an E value cut-off of 1e-8. Additional BLASTP searches were carried out against the NCBI nr database to retrieve any additional archaeal SelB (aSelB) -like sequences from incomplete genome projects. This also confirmed that aSelB-like sequences are only found within archaea. The identity of homologs as close relatives of SelB, rather than other paralogs within the translational GTPase (trGTPase) superfamily was verified by scanning against the translational GTPase database [46]. The resulting "aSelB" data set contained 60 sequences from 47 archaea. To root the aSelB phylogeny, 30 additional sequences from bacterial SelB, elongation factor Tu (EF-Tu) and archaeal initiation factor 2γ (aIF2γ) were retrieved from the translational GTPase database [46] and added to the data set.

Sequences were aligned using MAFFT v6.234b with strategy L-INS-I [47] for use in phylogenetic analyses. Poorly aligned and gap-rich regions of the alignment were identified by eye and excluded from the analyses. The 60 sequence aSelB and 90 sequence aSelB plus outgroup datasets consisted of 311 and 298 aligned amino acid columns, respectively. Maximum likelihood (ML) phylogenetic analysis was carried out with RAxML v 7.0.4 [48] on the CIPRES portal v2.2 http://www.phylo.org/sub_sections/portal/. The program was run with the PROTCATWAG model, with 100 bootstrap replicates. MrBayes v3.1.2 [49], also on the CIPRES portal v2.2, was run for 2 million generations, sampling every 100 generations, with 2 parallel runs of 8 chains each, using a gamma rate distribution with a mixed model that converged on WAG with a posterior probability of 0.99 for both data sets. A consensus tree was generated after discarding the first 200,000 generations from each run as a burn in. The standard deviation of split frequencies (SDSF) is shown in figure legends.

Genomic context was retrieved from the Entrez gene database [50], where records were available or via the CDS records within the NCBI genome entry. Protein structures were visualized and aligned with MacPyMOL.

Cysteine content was determined for archaeal predicted proteomes downloaded in Fasta format from the NCBI genome page http://www.ncbi.nlm.nih.gov/genomes/lproks.cgi. The percentage of cysteine in each proteome was calculated using a Python script.

## Authors' contributions

GCA conceived of the study, carried out sequence searching and phylogenetic analyses, and drafted the manuscript. VH participated in drafting the manuscript and in the analysis of cysteine content distribution. TT helped draft the manuscript and participated in the coordination of the study. All authors read and approved the final manuscript.

## Supplementary Material

Additional file 1 **Baysian inference tree of aSelB and aSelBL with aIF2γ, bacterial SelB and EF-Tu as the outgroup**. The tree was generated from 298 aligned amino acid positions. The SDSF at the end of the run was 0.02 Branch and tip labels are as per the Figure 1 legend. Click here for file

Additional file 2 **Table showing distribution of aSelBL, aSelB, SelD and pylS across complete archaeal genomes**. Square brackets indicate divergent or partial sequences (details in text). The percentage cysteine content of each proteome is indicated. Click here for file

Additional file 3 **Table of upstream and downstream genes from aSelBL and aSelB**. Arrows ">" show the direction of gene relative *aSelBL *or *aSelB*. The "X" at position 0 indicates the *aSelBL *or *aSelB *gene. HP stands for hypothetical protein, and is followed by additional details if it shares homology with a particular protein or domain. Genes with names in bold are transcribed in the same direction as aSelBL. Colours show homologous conserved flanking genes. Click here for file

Additional file 4 **Analysis of SelBL distribution corellation with cysteine content in archaeal proteomes**. Colored circles represent data points for individual genomes (see Additional file 2), and the colour code for taxonomic groups is given in the inset box. Click here for file
