# Supplementary figures and images for: An ancient family of SelB elongation factor-like proteins with a broad but disjunct distribution across archaea
Source: BMC Evol Biol. 2011 Jan 21;11:22. doi: 10.1186/1471-2148-11-22 (PMC3037878; doi:10.1186/1471-2148-11-22)

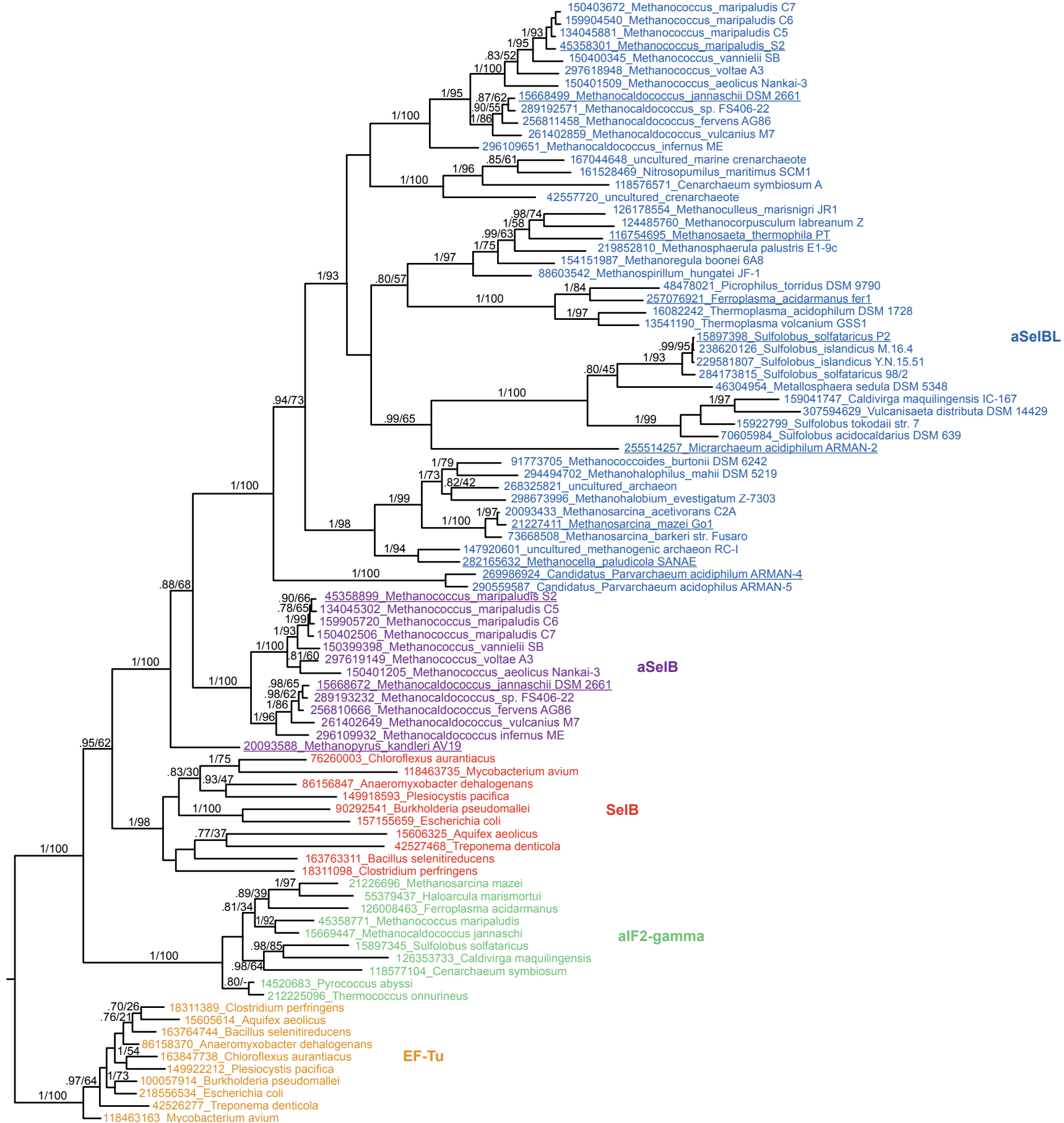

Supplement: Additional file 1 — Baysian inference tree of aSelB and aSelBL with aIF2γ, bacterial SelB and EF-Tu as the outgroup. The tree was generated from 298 aligned amino acid positions. The SDSF at the end of the run was 0.02 Branch and tip labels are as per the Figure 1 legend. [file 1471-2148-11-22-S1.PDF]

aSelBL presence

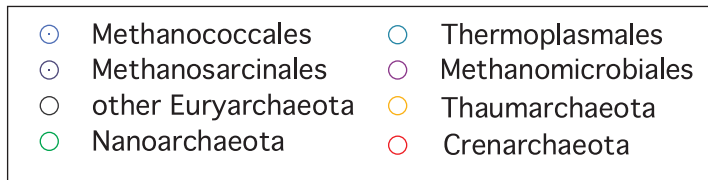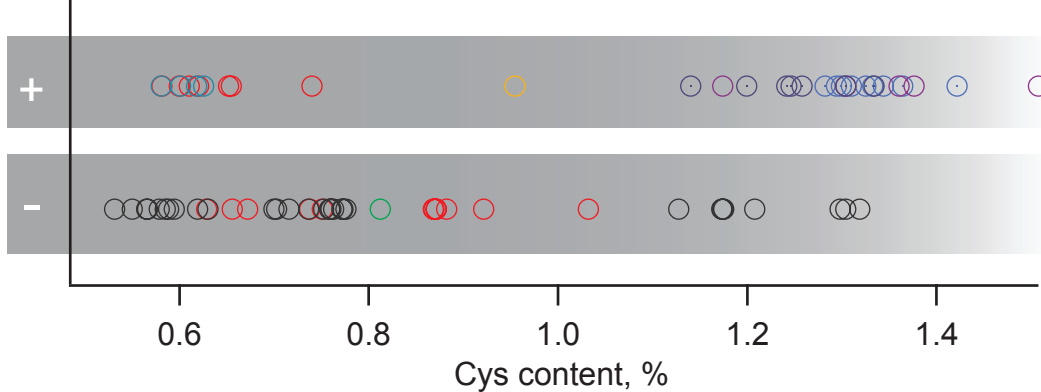

Supplement: Additional file 4 — Analysis of SelBL distribution corellation with cysteine content in archaeal proteomes. Colored circles represent data points for individual genomes (see Additional file 2), and the colour code for taxonomic groups is given in the inset box. [file 1471-2148-11-22-S4.PDF]
